# Geographic variation in kidney failure and transplantation in Aotearoa New Zealand: A population‐based data linkage study

**DOI:** 10.1111/nep.14409

**Published:** 2024-10-28

**Authors:** Johanna M. Birrell, Angela C. Webster, Nicholas B. Cross, Tim Driscoll, Heather Dunckley, Ben Beaglehole, Ian Dittmer, Curtis Walker, Merryn Jones, John Irvine, Melanie L. Wyld, Kate R. Wyburn, Nicole L. De La Mata

**Affiliations:** ^1^ Department of Nephrology Te Whatu Ora Waitaha Canterbury Christchurch New Zealand; ^2^ School of Public Health University of Sydney Sydney New South Wales Australia; ^3^ Department of Renal Medicine Westmead Hospital Sydney New South Wales Australia; ^4^ New Zealand Blood Service Auckland New Zealand; ^5^ Department of Psychological Medicine University of Otago Christchurch New Zealand; ^6^ Department of Renal Medicine, Auckland City Hospital Te Whatu Ora Te Toka Tumai Auckland New Zealand; ^7^ Medical Council of New Zealand, Te Whatu Ora Te Pae Hauora o Ruahine o Tararua MidCentral Palmerston North New Zealand; ^8^ Kidney Health New Zealand Tākihi Hauora Aotearoa Christchurch New Zealand; ^9^ Department of Renal Medicine Royal Prince Alfred Hospital Sydney New South Wales Australia

**Keywords:** epidemiology, geographic information systems, health equity, kidney transplantation, New Zealand

## Abstract

**Aim:**

This study aimed to describe the epidemiology of kidney replacement therapy (KRT) in Aotearoa New Zealand and assess the impact of residential location on access to kidney transplantation.

**Methods:**

AcceSS and Equity in Transplantation (ASSET), a health‐linked data platform, was used to identify people commencing KRT in New Zealand from 2006 to 2019 and analyse regional epidemiology. Health services were classified as ‘transplanting’, ‘intermediate’ or ‘remote’ depending on their degree of separation from a transplant centre. Multiple logistic regression modelling was used to assess the predictors of deceased donor waitlisting or living donor transplantation within 6 months after starting KRT. Web‐based mapping software was used to develop interactive geospatial maps.

**Results:**

The cohort was 7704 people newly starting KRT. Living in an intermediate [odds ratio (OR): 0.73 (95% confidence interval (CI): 0.61–0.88)] or remote [OR: 0.38 (95% CI: 0.27–0.54)) region and Māori (OR: 0.35 (95% CI: 0.28–0.44)], Pacific [OR: 0.32 (95% CI: 0.24–0.42)) and Asian (OR: 0.66 (95% CI: 0.50–0.87)] ethnicity were associated with a decreased likelihood of timely waitlisting or transplantation. Regional maps can be explored here.

**Conclusion:**

There is marked geospatial and ethnic variation in the epidemiology of kidney failure and access to kidney transplantation across New Zealand. Geospatial mapping of kidney failure epidemiology and transplantation outcomes can provide opportunities to direct resources towards populations at greatest need.

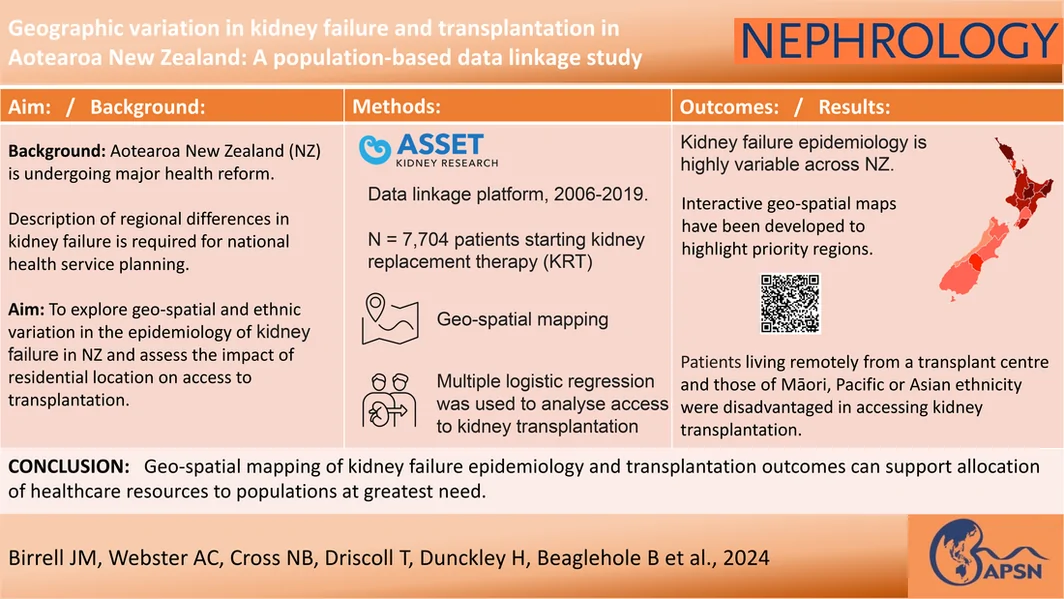

## Summary at a Glance

This population‐based data linkage study describes marked spatial, temporal and ethnic variation in the epidemiology of kidney failure across Aotearoa New Zealand. People living remotely from a transplant centre and those of Māori, Pacific or Asian ethnicity were found to be independently disadvantaged in accessing kidney transplantation.

## 1 INTRODUCTION

Kidney failure is an increasingly important public health issue in Aotearoa New Zealand. In 2022, 5474 people received kidney replacement therapy (KRT) in New Zealand – 3157 on dialysis and 2317 living with a kidney transplant.^1^ The number of people receiving KRT has increased by more than fourfold (from 1344 people) over the past 30 years.^1^

With an ageing population, the burden of kidney failure is anticipated to rise dramatically over coming years.^2^ The annual cost of dialysis per person in New Zealand has been estimated at $115 712 per year in 2021 New Zealand dollars.^2^ Kidney transplantation is considered the treatment of choice for most people with kidney failure, with survival and quality of life advantages, and is more cost‐effective than dialysis.^2^, ^3^, ^4^ However, health system organization may create inequities in access to optimal kidney care.

Access to kidney transplantation in New Zealand may be affected by geographic proximity to health services. Living remotely from a transplant centre has been associated with reduced access to kidney transplantation in other countries,^5^, ^6^, ^7^ but this has not been quantitatively studied in New Zealand.

New Zealand has a public health care system funded through general taxation, with a private system providing predominantly surgical and diagnostic procedures.^8^ All New Zealand citizens and permanent residents have access to government‐funded KRT, so almost all dialysis and transplantation take place in the public sector. The New Zealand Kidney Allocation Scheme provides an algorithm for nationwide allocation of all deceased donor and non‐directed living donor kidneys.^9^

New Zealand is currently undergoing major health reform, with a central focus on creating an equitable and cohesive national health service. On 1 July 2022, New Zealand dis‐established its former 20 district health boards (DHBs) and transitioned to a national health governance body, Te Whatu Ora – Health New Zealand.^10^ Extensive health service planning is required to improve patient access to kidney failure services around the country and prepare for future demand. An evidence‐based approach requires detailed regional epidemiologic data to ensure that under‐served population groups are identified and receive additional health support.

The AcceSS and Equity in Transplantation (ASSET) project has created a linked data platform to facilitate research enquiry into equity of health service delivery for people with kidney failure in New Zealand.^11^ Using the ASSET platform, this study aimed to describe geographic variation in the epidemiology of KRT around New Zealand and assess the impact of residential location on access to kidney transplantation.

## 2 METHODS

### 2.1 Data sources and linkage

A cohort of people newly starting KRT in New Zealand between 2006 and 2019 was defined using ASSET, drawing on data from the Australia and New Zealand Dialysis and Transplant Registry (ANZDATA). Variables extracted from ANZDATA included a unique identification number, age, sex, ethnicity, comorbidities, primary renal disease, smoking status, body mass index (BMI), KRT start date, initial KRT type, late referral status, transplantation date and death date (if applicable). ‘Late referral’ was defined as the first assessment by a specialist nephrologist occurring within 3 months of commencing KRT. Ethnicity categories available were recoded to align with Stats NZ categories.^12^ The ‘Middle Eastern/Latin American/African’ category was combined with ‘other ethnicity’ due to small numbers. It was possible for multiple ethnicities to be recorded for a single patient (total response ethnicity).^12^

**FIGURE 1:**
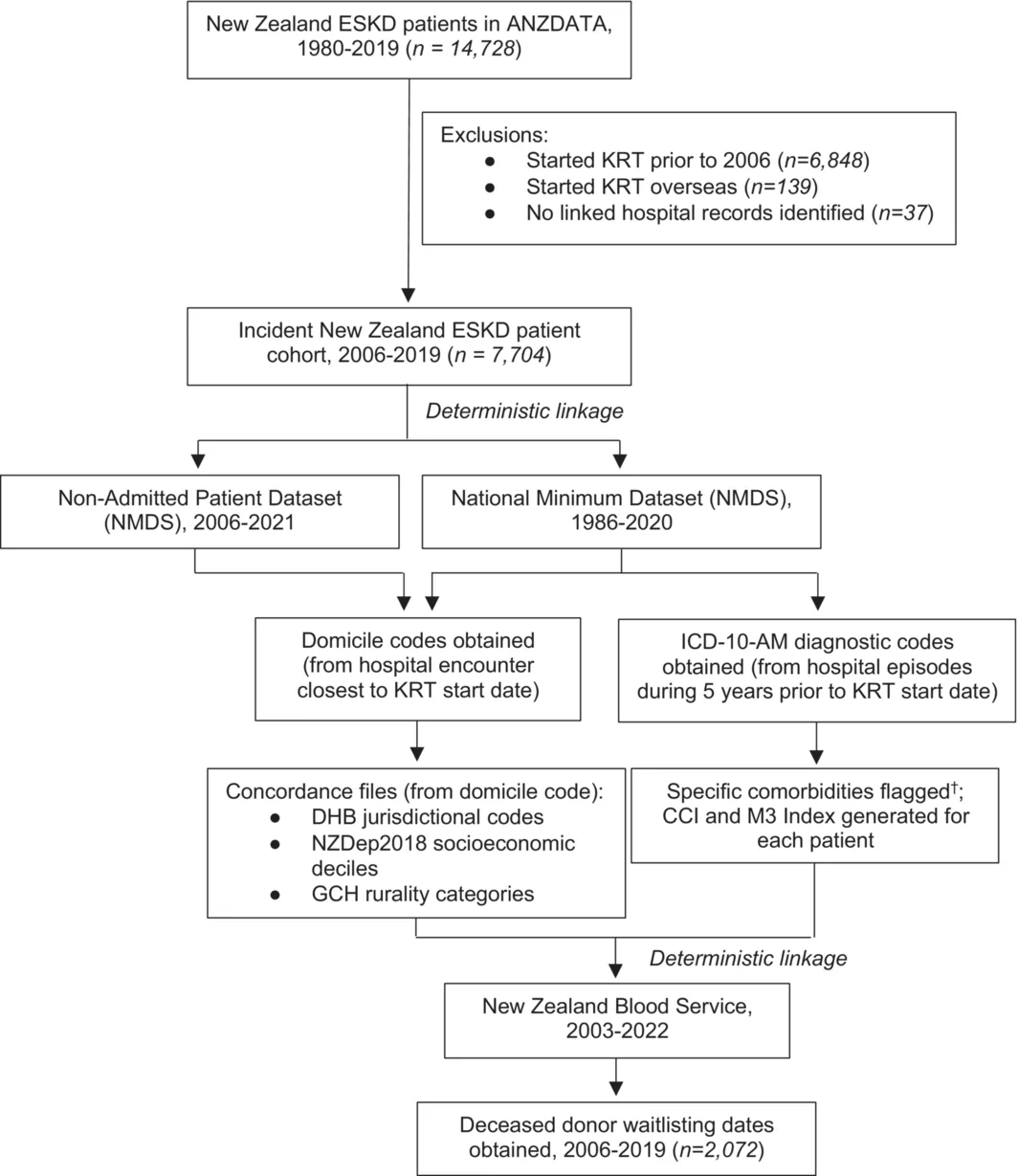
Flowchart of data linkage process. Records were included from the AcceSS and Equity in Transplantation (ASSET) data platform, derived from the Australia and New Zealand Dialysis and Transplant Registry, National Minimum Dataset, Non‐Admitted Patient Dataset and New Zealand Blood Service. ^†^Diabetes mellitus, chronic lung disease, coronary artery disease, peripheral vascular disease, cerebrovascular disease, cancer. ANZDATA, Australia and New Zealand Dialysis and Transplant Registry; CCI, Charlson Comorbidity Index; DHB, District Health Board; ESKD, end stage kidney disease; GCH, Geographic Classification for Health; ICD‐10‐AM, International Classification of Diseases and Related Health Problems, Tenth Revision, Australian Modification; KRT, kidney replacement therapy; M3 Index, M3 Multimorbidity Index; NZDep2018, New Zealand Index of Deprivation, 2018.

For each member of the cohort, corresponding records were deterministically linked to the National Minimum Dataset (NMDS), National Non‐Admitted Patient Data Collection (NNPAC) and New Zealand Blood Service (NZBS) Database (Figure 1). Linkage was performed using encrypted National Health Index (NHI) numbers, via the ASSET Platform. The NMDS is a national collection of public and private hospital discharge information. Variables extracted from the NMDS included domicile code (obtained from information recorded for the hospital admission date temporally closest to each patient's KRT start date) and International Classification of Diseases and Related Health Problems, Tenth Revision, Australian Modification (ICD‐10‐AM) diagnostic codes for the 5 years preceding the KRT start date. Domicile codes, a tool used by the Ministry of Health, were used to represent a person's usual residential address. These codes are derived from Stats NZ Census Area Units and contiguous domicile codes form DHB areas.^13^ The NNPAC was used as a secondary source to identify domicile codes for patients with a missing domicile in the NMDS. The NZBS database provided dates of initial deceased donor transplant waitlisting.

Domicile codes were linked to DHB regions, Rural–Urban Geographic Classification for Health (GCH) codes and New Zealand Index of Deprivation 2018 (NZDep2018) socioeconomic deciles using concordance files.^14^, ^15^, ^16^ The NZDep2018 score is the standard approach in New Zealand for estimating relative socioeconomic advantage or disadvantage. NZDep2018 combines variables from the 2018 census, incorporating eight dimensions of socioeconomic deprivation (communication, income, employment, qualifications, owned home, support, living space and living condition). A score from 1 to 10 (where 1 is least disadvantaged) is produced for each statistical area geographical region in New Zealand.^16^

**FIGURE 2:**
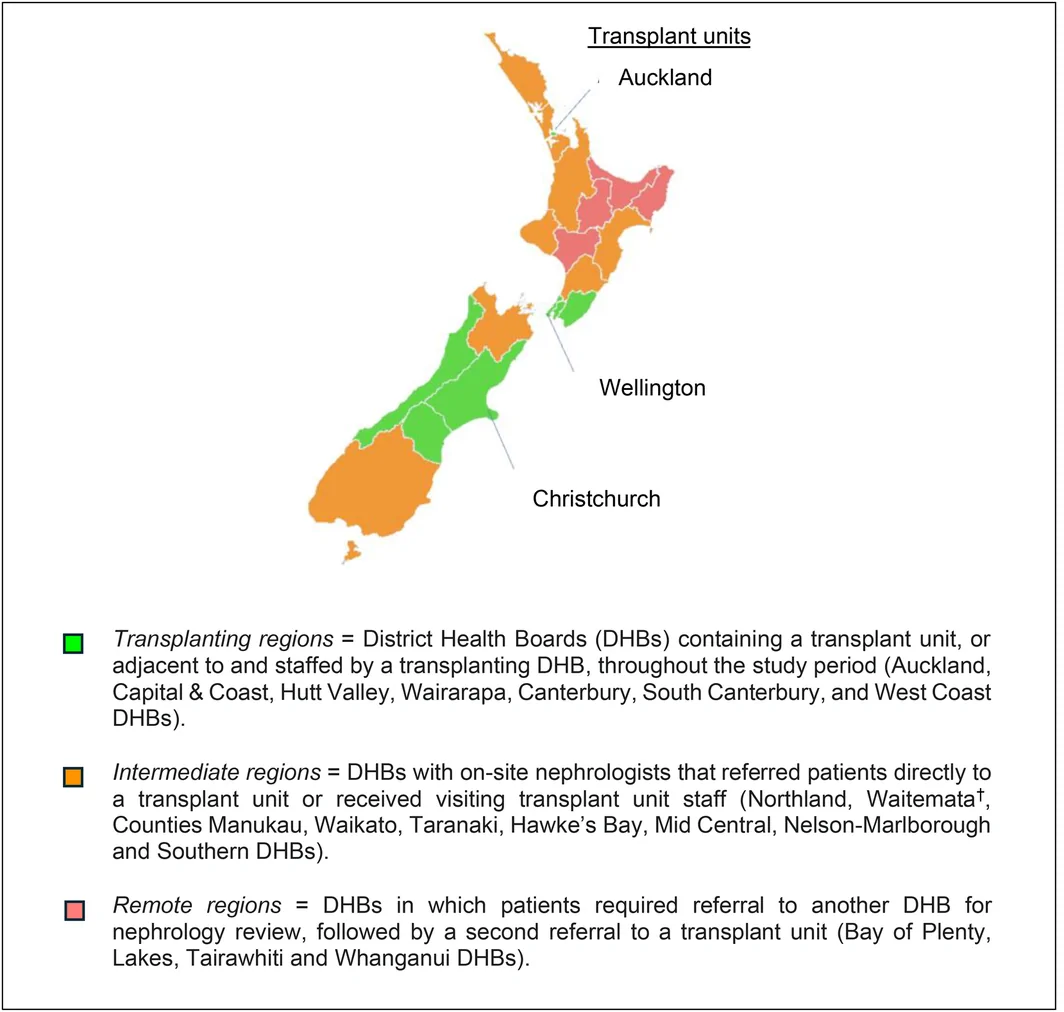
Regional kidney transplantation categories, by District Health Board. Regions were classified as transplanting, intermediate or remote depending on the degree of nephrology service separation from a kidney transplant unit. ^†^Waitemata DHB changed from ‘transplanting’ to ‘intermediate’ in 2011 but was classified as ‘intermediate’ for the purposes of this study.

DHBs were categorized as transplanting, intermediate or remote depending on the degree of nephrology service separation from a transplant unit. Transplanting regions were defined as DHBs containing a kidney transplant unit, or adjacent to and staffed by a transplanting DHB, throughout the study period. Intermediate regions were defined as DHBs with on‐site specialist nephrologists that referred patients directly to a transplant unit or received visiting transplant unit staff. Remote regions were defined as DHBs in which patients required referral to another DHB for specialist nephrology review, followed by a second referral to a transplant unit (Figure 2).

### 2.2 Incidence calculations

Annualized population data from Stats NZ, in 5‐year age bands, by sex and DHB, were used for incidence calculations.^17^ Direct age standardization was performed using the World Health Organization Standard Population Distribution as the reference population, to allow for international incidence comparisons.^18^

### 2.3 Statistical analysis

Statistical analyses were undertaken in RStudio® (Version 2023.03.0+386) and Stata® (Version 14.2). Patient characteristics were summarized using absolute numbers and proportions. ICD‐10‐AM codes from the NMDS were used to identify selected diagnoses (diabetes mellitus, chronic lung disease, coronary artery disease, peripheral vascular disease, cerebrovascular disease and cancer) to validate against ANZDATA comorbidity data. Agreement was assessed based on whether each condition was identified in both the ANZDATA and NMDS data sets, using Cohen's kappa statistic (*k*).^19^, ^20^ Agreement was described using Landis and Koch's qualitative assessment of the *k* statistic.^21^ Charlson Comorbidity Index (CCI) and M3 Multimorbidity Index were calculated for each patient using ICD‐10‐AM codes, with renal disease excluded from the scoring criteria.^22^, ^23^

Within each DHB jurisdiction, we calculated the proportion of the cohort that were: (1) living in the lowest socioeconomic residential quintile, (2) living in a rural location (GCH category ‘Rural1’, ‘Rural2’ or ‘Rural3’) and (3) had a high level of comorbidity (CCI of two and above) upon starting KRT.

The Australian and New Zealand Society of Nephrology (ANZSN) has a key performance indicator (KPI) of deceased donor waitlisting or transplantation within 6 months of KRT commencement, for people aged 2–64 years.^24^ The status of this outcome was calculated for each member of the cohort. Patients aged under 2 or over 64 years at commencement of KRT were excluded from this analysis.

Multiple logistic regression analysis was performed for the outcomes of (1) deceased donor waitlisting, (2) living donor transplantation or (3) either of the two, within 6 months of KRT commencement. Results were presented as odds ratios (ORs) and 95% confidence intervals (CIs). Age and BMI were recoded into clinically relevant categories. Predictor variables were considered for inclusion in the multiple logistic regression model if significant on univariate analysis with a *p*‐value threshold of less than .05, using a backward elimination approach.

The following patient‐level variables were included in the model: age (2–45, 45–54, 55–64 years), sex, socioeconomic index (NZDep2018 quintile), ethnicity (European, Māori, Pacific, Asian, other ethnicity), BMI category, M3 Multimorbidity Index, late referral, year of starting KRT (2006–10, 2011–15, 2016–19) and transplanting region category (Figure 2). Sex did not reach the *p*‐value threshold but was retained as it was considered an important predictor of the outcomes.^25^ Rurality and CCI were excluded from the model as they were highly correlated with other included variables (transplanting region category and M3 Multimorbidity Index respectively). The M3 Multimorbidity Index was selected for inclusion as studies have demonstrated that it outperformed CCI in a New Zealand context. A higher M3 Index score (possible range ≥0, with a 99th percentile of 1.93) indicates a greater level of multimorbidity.^23^ Model fit was assessed using the McFadden's R‐squared test, c statistic and Hosmer–Lemeshow test. Esri ArcGIS® software was used to develop interactive geospatial maps.

### 2.4 Ethics approval

The ASSET project, including this study, received ethics approval from the University of Sydney (HREC 2020/871). The Health and Disability Ethics Committee, New Zealand, determined that the ASSET project was out of scope for ethics review due to the use of de‐identified data with approval not required.

## 3 RESULTS

### 3.1 Cohort characteristics

A total of 7704 incident KRT recipients were included in the analysis. Table 1 shows the characteristics of the cohort. The median age was 58 years (interquartile range (IQR) 48–68) and 60% of patients were male. Māori and Pacific people were over‐represented, comprising 31% and 21% of the KRT cohort, respectively (compared to 17% and 8% of the overall New Zealand population) (Figure S1). Diabetes was the cause of kidney failure for 68% (*n* = 1628) of Māori and 68% (*n* = 1094) of Pacific patients, compared to 20% (*n* = 608), 47% (*n* = 298) and 34% (*n* = 25) for patients of European, Asian and other ethnicity groups, respectively. People commencing KRT were more likely to be in the most socioeconomically disadvantaged quintile (41% in NZDep2018 decile 9–10 areas), in contrast to the general population of New Zealand (21%). However, rurality was similar in the KRT cohort to the background population (Figure S1).

**TABLE 1:** Characteristics of incident patients starting kidney replacement therapy in New Zealand from 2006 to 2019, by region category^a^. Data are numbers (%).

Characteristics	Transplanting regions (*n* = 2136)	Intermediate regions (*n* = 4690)	Remote regions (*n* = 850)	Total (*n* = 7704)
Deaths during follow‐up	974 (46)	2257 (48)	458 (54)	3702 (48)
Sex				
Male	1279 (60)	2796 (60)	541 (64)	4633 (60)
Female	857 (40)	1894 (40)	309 (36)	3071 (40)
Age at kidney failure, years				
≤17	40 (2)	89 (2)	7 (1)	137 (2)
18–34	174 (8)	354 (8)	57 (7)	587 (8)
35–44	215 (10)	429 (9)	76 (9)	727 (9)
45–54	459 (21)	965 (21)	197 (23)	1628 (21)
55–64	554 (26)	1278 (27)	235 (28)	2072 (27)
65–74	485 (23)	1116 (24)	216 (25)	1823 (24)
≥75	209 (10)	459 (10)	62 (7)	730 (9)
Year of kidney failure				
2006–2010	693 (32)	1558 (33)	292 (34)	2551 (33)
2011–2015	774 (36)	1637 (35)	258 (30)	2679 (35)
2016–2019	669 (31)	1495 (32)	300 (35)	2474 (32)
Ethnicity^b^				
European	950 (44)	1731 (37)	296 (35)	2979 (39)
Māori	327 (15)	1550 (33)	500 (59)	2381 (31)
Pacific	547 (26)	1012 (22)	29 (3)	1609 (21)
Asian	286 (13)	340 (7)	17 (2)	644 (8)
Middle Eastern/Latin American/African	19 (1)	37 (1)	1 (0.1)	57 (1)
Other ethnicity	7 (0.3)	9 (0.2)	4 (0.5)	20 (0.3)
Not collected	5 (0.2)	21 (0.4)	4 (0.5)	30 (0.4)
Socioeconomic quintile				
1–2 (least disadvantaged)	293 (14)	354 (8)	39 (5)	686 (9)
3–4	367 (17)	482 (10)	73 (9)	922 (12)
5–6	399 (19)	688 (15)	104 (12)	1191 (16)
7–8	547 (26)	980 (21)	158 (19)	1685 (22)
9–10 (most disadvantaged)	530 (25)	2183 (47)	476 (56)	3189 (41)
Rurality^c^				
Urban 1	1861 (87)	2797 (60)	229 (27)	4888 (64)
Urban 2	105 (5)	905 (19)	373 (44)	1383 (18)
Rural 1	139 (7)	530 (11)	169 (20)	838 (11)
Rural 2	24 (1)	381 (8)	50 (6)	455 (6)
Rural 3	6 (0.3)	74 (2)	29 (3)	109 (1)
Cause of kidney failure				
Diabetes	928 (43)	2267 (48)	452 (53)	3661 (48)
Glomerulonephritis	488 (23)	914 (19)	165 (19)	1569 (20)
Hypertension or renal artery disease	203 (10)	455 (10)	72 (8)	734 (10)
Polycystic kidney disease	127 (6)	219 (5)	42 (5)	389 (5)
Reflux nephropathy	60 (3)	86 (2)	16 (2)	162 (2)
Other	240 (11)	561 (12)	74 (9)	879 (11)
Uncertain diagnosis	87 (4)	171 (4)	26 (3)	287 (4)
Not reported	3 (0.1)	17 (0.4)	3 (0.4)	23 (0.3)
Initial kidney replacement therapy (KRT) modality				
Haemodialysis – in‐centre	1260 (59)	2917 (62)	485 (57)	4685 (61)
Haemodialysis – home	29 (1.4)	42 (0.9)	5 (0.6)	76 (1.0)
Peritoneal dialysis	705 (33)	1564 (33)	343 (40)	2617 (34)
Pre‐emptive transplantation	142 (7)	167 (4)	17 (2)	326 (4)
KRT modality at 1 year after commencement				
Haemodialysis – in‐centre	790 (37)	1763 (38)	184 (22)	2749 (36)
Haemodialysis – home	182 (9)	344 (7)	74 (9)	602 (8)
Peritoneal dialysis	714 (33)	1810 (39)	467 (55)	2998 (39)
Transplantation	241 (11)	296 (6)	32 (4)	570 (7)
Death	173 (8)	398 (8)	79 (9)	654 (8)
Recovered renal function	30 (1)	69 (1)	14 (2)	113 (1)
Lost to follow up	6 (0.3)	10 (0.2)	0 (0)	18 (0.2)
Patients starting haemodialysis with an arteriovenous fistula/graft, or starting on peritoneal dialysis^d^	1106 (55)	2346 (52)	454 (55)	3912 (53)

^a^ Region category was unable to be allocated from domicile code for 28 patients.

^b^ Categorized based on Stats NZ ethnic groups, using total response ethnicity.^12^

^c^ Rurality was unable to be allocated from domicile code for 31 patients.

^d^ Denominator is the total number of patients starting kidney replacement therapy with dialysis.^23^

In New Zealand overall, the most common initial KRT modality was in‐centre (hospital or satellite) haemodialysis (HD; 61%), followed by peritoneal dialysis (PD; 34%), pre‐emptive transplantation (4%) and home HD (1%). At 1 year after KRT commencement, PD was the most frequent modality (39%), followed by in‐centre HD (36%), home HD (8%) and kidney transplantation (7%). As outlined in Table 1, patients living in remote regions were more likely to be receiving PD at 1 year (55%) than patients in intermediate (39%) and transplanting regions (33%). Remote patients had a lower rate of transplantation (4%) and in‐centre HD (22%) at 1 year when compared to other region categories. The overall national mortality rate within 1 year of starting KRT was 8%.

Table 2 shows the comorbidity characteristics of people starting KRT, by transplanting region category. Patients living in intermediate and remote regions had a higher burden of multimorbidity and smoking (at any time) than those in transplanting regions. The median M3 Multimorbidity Index score was 0.97 (IQR 0.48–1.55) for Māori patients and 0.84 (IQR 0.46–1.39) for Pacific patients, compared to 0.54 (IQR 0.18–1.22) for European, 0.64 (IQR 0.27–1.17) for Asian and 0.67 (IQR 0.27–1.13) for other ethnicity groups. Tables S1 and S2 and Figure S2 outline the correlation between comorbidity indices used in this study, as derived separately from ANZDATA and NMDS ICD‐10‐AM diagnostic codes.

**TABLE 2:** Comorbidity status of incident patients starting kidney replacement therapy in New Zealand from 2006 to 2019, by region category^a^. Data are numbers (%) unless stated otherwise.

Comorbidity characteristics	Transplanting regions (*n* = 2136)	Intermediate regions (*n* = 4690)	Remote regions (*n* = 850)	Total (*n* = 7704)
Comorbidity at kidney failure (from ANZDATA)				
Diabetes	1079 (51)	2632 (56)	495 (58)	4222 (55)
Coronary heart disease	487 (23)	1217 (26)	214 (25)	1924 (25)
Chronic lung disease	217 (10)	703 (15)	144 (17)	1066 (14)
Peripheral vascular disease	219 (10)	653 (14)	106 (12)	978 (13)
Cerebrovascular disease	208 (10)	493 (11)	62 (7)	764 (10)
Malignancy (history of)	269 (13)	544 (12)	83 (10)	898 (12)
Count of above comorbidities (from ANZDATA)				
0	682 (32)	1171 (25)	203 (24)	2064 (27)
1	748 (35)	1679 (36)	330 (39)	2771 (36)
≥2	706 (33)	1840 (40)	317 (37)	2869 (38)
Charlson Comorbidity Index^b^				
0	750 (35)	1357 (29)	238 (28)	2353 (31)
1	167 (8)	329 (7)	63 (7)	559 (7)
≥2	1208 (57)	2982 (64)	548 (64)	4753 (62)
Unable to be calculated	11 (0.5)	22 (0.5)	1 (0.1)	39 (0.5)
M3 Multimorbidity Index^b^				
0	262 (12)	471 (10)	105 (12)	842 (11)
>0–1.0	1105 (52)	2316 (49)	391 (46)	3827 (50)
>1.0	755 (35)	1871 (40)	352 (42)	2982 (39)
Unable to be calculated	14 (0.7)	32 (0.7)	2 (0.2)	53 (0.7)
Smoking status				
Current	284 (13)	702 (15)	152 (18)	1141 (15)
Former	735 (34)	1883 (40)	352 (41)	2981 (39)
Never	1114 (52)	2019 (43)	341 (40)	3487 (45)
Not collected	3 (0.1)	86 (2)	5 (0.6)	95 (1)
Body mass index				
Underweight (≤18.4)	54 (3)	103 (2)	9 (1)	166 (2)
Normal (18.5–24.9)	555 (26)	950 (20)	169 (20)	1680 (22)
Overweight (25.0–29.9)	644 (30)	1288 (27)	225 (26)	2164 (28)
Obese (≥30.0)	875 (41)	2133 (45)	424 (50)	3446 (45)
Not collected	8 (0.4)	216 (5)	23 (3)	248 (3)

^a^ Region category was unable to be allocated from domicile code for 28 patients.

^b^ Kidney failure parameters were excluded from scoring systems.

### 3.2 Incidence of KRT

**FIGURE 3:**
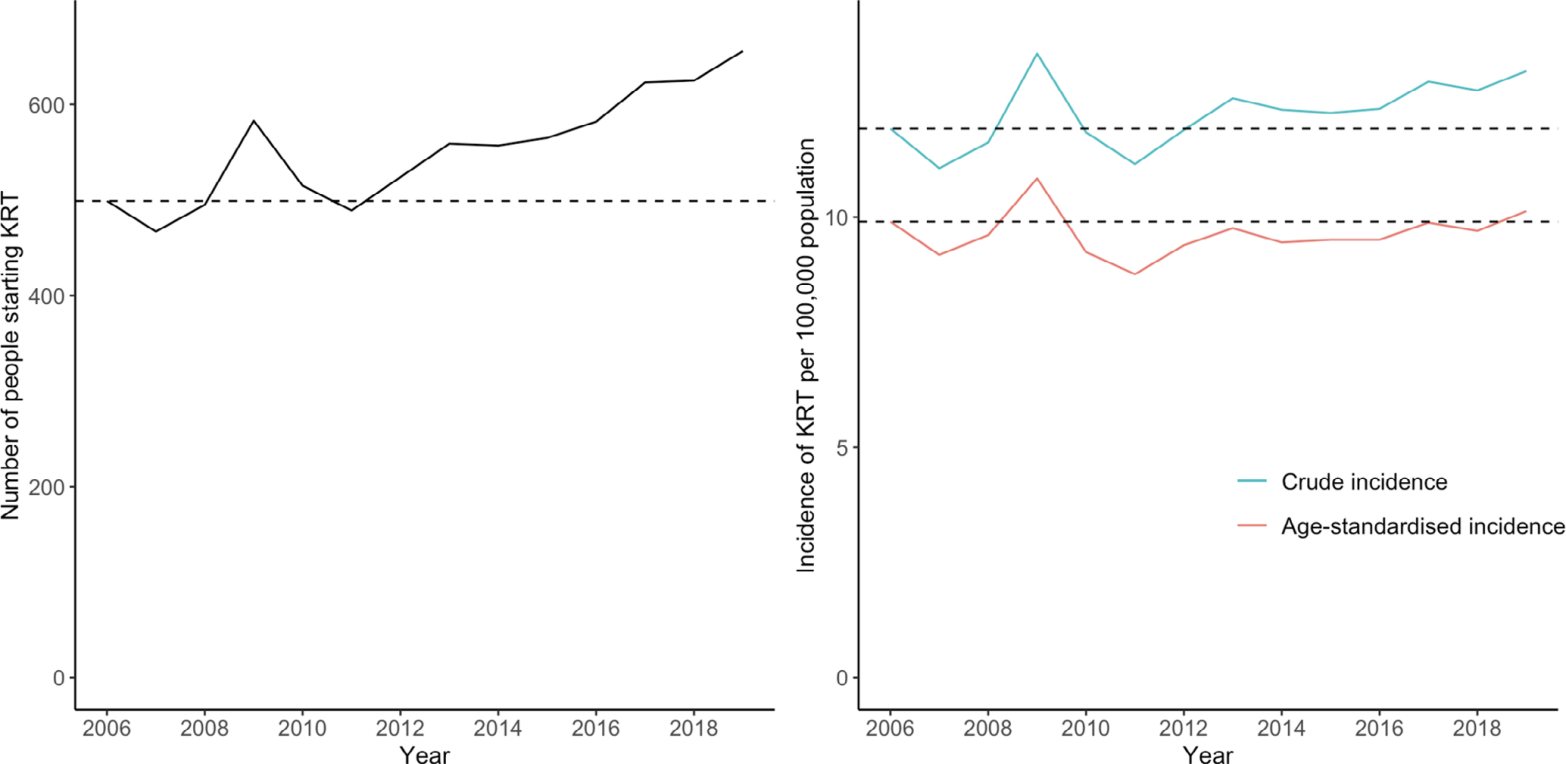
Trend in number of patients, crude and age‐standardized incidence of kidney replacement therapy over time, New Zealand, 2006–2019. KRT, kidney replacement therapy.

The overall age‐standardized incidence of KRT in New Zealand during the study period was 9.6 (95% CI 9.4–9.8) cases per 100 000 population and remained fairly stable over the study period (Figure 3). There was a higher incidence of KRT in males than females, at 11.8 (95% CI 11.5–12.2) and 7.6 (95% CI 7.3–7.9) cases per 100 000 population, respectively. The mean annualized population over the study period was largest across intermediate regions (*n* = 2 513 825), followed by transplanting (*n* = 1 553 583) and remote regions (*n* = 438 500). The incidence of KRT was 8.0 (95% CI 7.7–8.3) cases per 100 000 population in transplanting regions, 10.3 (95% CI 10.0–10.7) in intermediate regions and 10.3 (95% CI 9.6–11.0) in remote regions.

### 3.3 Geographic variation in kidney failure epidemiology

**FIGURE 4:**
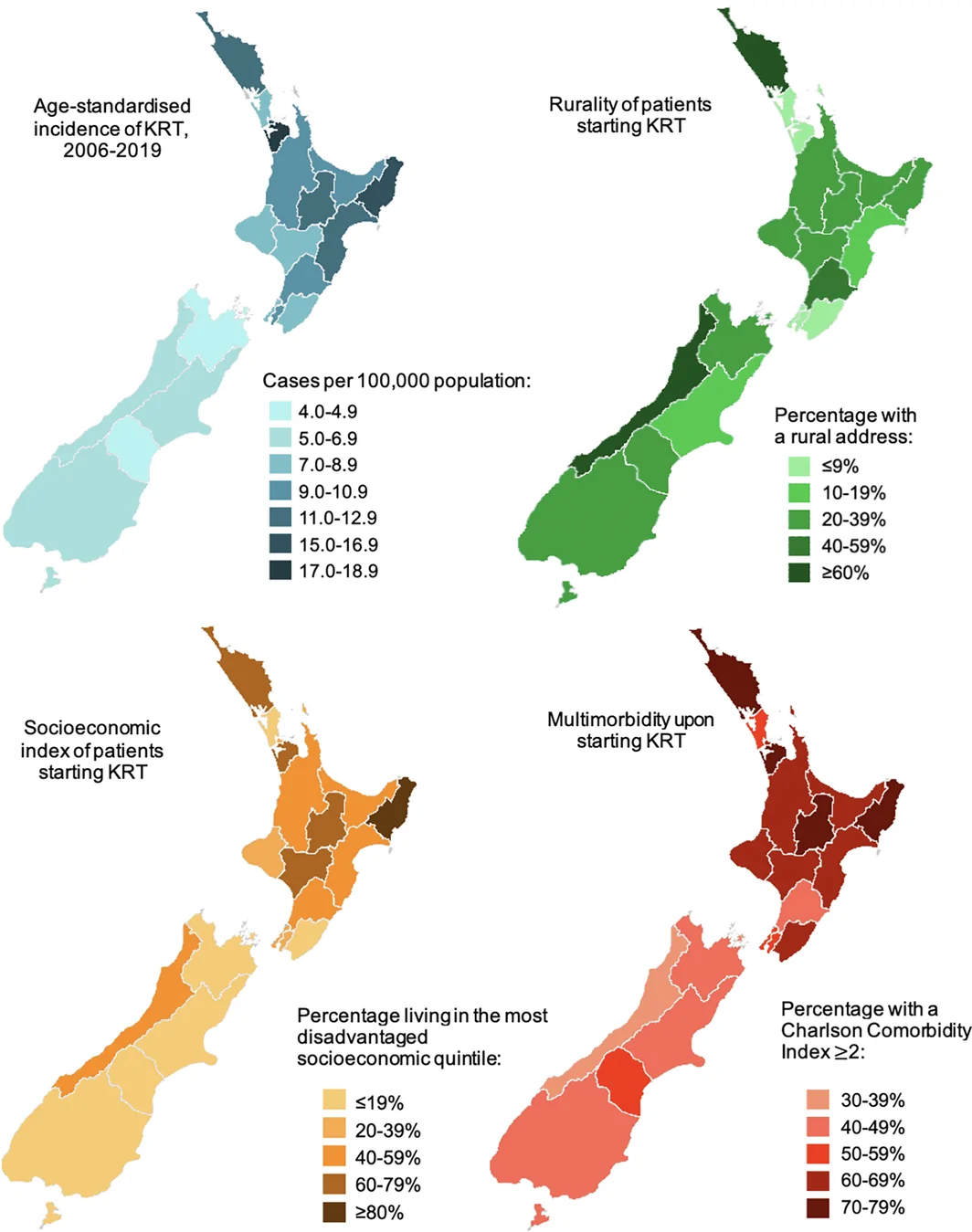
Geographic variation across former District Health Boards of New Zealand, with reference to age‐standardized incidence of kidney replacement therapy (KRT) (top‐left) and rurality (top‐right), socioeconomic index (bottom‐left) and multimorbidity (bottom‐right) of patients starting KRT from 2006 to 2019. Rural address defined as Geographic Classification for Health R1–R3 area, based on domicile. Using NZDep2018 Index, based on domicile. Charlson Comorbidity Index of four or higher (after additional two points are added for severe chronic kidney disease) has been estimated to predict a 10‐year survival probability of less than 54%.^22^

Figure 4 illustrates the significant geospatial variation (by DHB) in incidence of KRT, ranging from 4.6 (95% CI 3.7–5.6) cases per 100 000 in Nelson‐Marlborough to 18.5 (95% CI 17.5–19.4) in Counties Manukau. Tairawhiti, Northland, Counties Manukau and Lakes DHBs recorded a high incidence of KRT, overlapping with high rates of socioeconomic disadvantage and multimorbidity. The DHBs with the highest proportion of patients with a rural address were West Coast and Northland. Further detail can be found in our interactive maps.

### 3.4 Waitlisting and transplantation rates

The median follow‐up time was 6.5 years (IQR 3.1–10.2). At completion of the study period, 2072 patients (27%) had been waitlisted and 1499 (19%) had received a kidney transplant (Table 3). A total of 3258 patients (42%) had died since starting dialysis, without prior waitlisting or transplantation (Table S3) and 329 patients (4%) died after waitlisting, without ever receiving a transplant. Within the subgroup never waitlisted, the median time from starting dialysis to death was 2.7 years (IQR 1.3–4.6).

**TABLE 3:** Waitlisting and transplantation outcomes for patients starting kidney replacement therapy in New Zealand during 2006–2019, by region category. Data are numbers (%) unless stated otherwise.

Outcome	Transplanting regions (*n* = 2136)	Intermediate regions (*n* = 4690)	Remote regions (*n* = 850)	Total (*n* = 7704)
Transplantation status				
Waitlisted during follow‐up	651 (30)	1222 (26)	193 (23)	2072 (27)
Transplanted during follow‐up	547 (26)	841 (18)	108 (13)	1499 (19)
Donor transplantation	246 (12)	411 (9)	52 (6)	711 (9)
Living donor transplantation	301 (14)	427 (9)	56 (7)	785 (10)
Donor type not recorded	0 (0)	3 (<0.1)	0 (0)	3 (<0.1)
Not waitlisted or transplanted during follow‐up	1341 (63)	3305 (70)	650 (76)	5316 (69)
Timing of waitlisting				
Waitlisted before starting KRT	231 (11)	382 (8)	56 (7)	669 (9)
Waitlisted after starting KRT	420 (20)	840 (18)	137 (16)	1404 (18)
Median time from starting dialysis to waitlisting, in months (IQR)^a^	10 (5–21)	14 (7–20)	18 (7–28)	13 (7–25)
Timing of transplantation				
Median time from starting KRT to living donor transplantation, in months (IQR)^b^	5 (0–17)	10 (0–24)	15 (0–27)	9 (0–22)
Median time from starting KRT to deceased donor transplantation, in months (IQR)^b^	30 (14–51)	36 (17–60)	28 (14–48)	34 (15–57)
Waitlisting or transplantation within 6 months after starting KRT^c^				
Waitlisted within 6 months	314 (22)	488 (16)	68 (12)	871 (17)
Living donor transplantation within 6 months	145 (10)	169 (5)	17 (3)	332 (6)
Waitlisting or living donor transplantation within 6 months	420 (29)	577 (19)	70 (12)	1069 (21)

^a^ Patients waitlisted prior to starting dialysis were excluded from this calculation.

^b^ Includes patients undergoing pre‐emptive transplantation (time to transplantation = 0 months).

^c^ Only including patients aged 2–64 years at commencement of KRT, as per ANZSN Key Performance Indicator. Denominators for this age group: transplanting regions: *n* = 1432; intermediate regions: *n* = 3102; remote regions: *n* = 572; all regions: *n* = 5128.

As shown in Table 3, patients living in a transplanting region had a higher overall rate of both waitlisting and transplantation than those living in intermediate and remote regions, and a shorter median time from starting KRT to waitlisting and living donor transplantation. Figure S3 shows the distribution of times from starting KRT to waitlisting by region category.

### 3.5 Logistic regression analysis of access to waitlisting or transplantation

**FIGURE 5:**
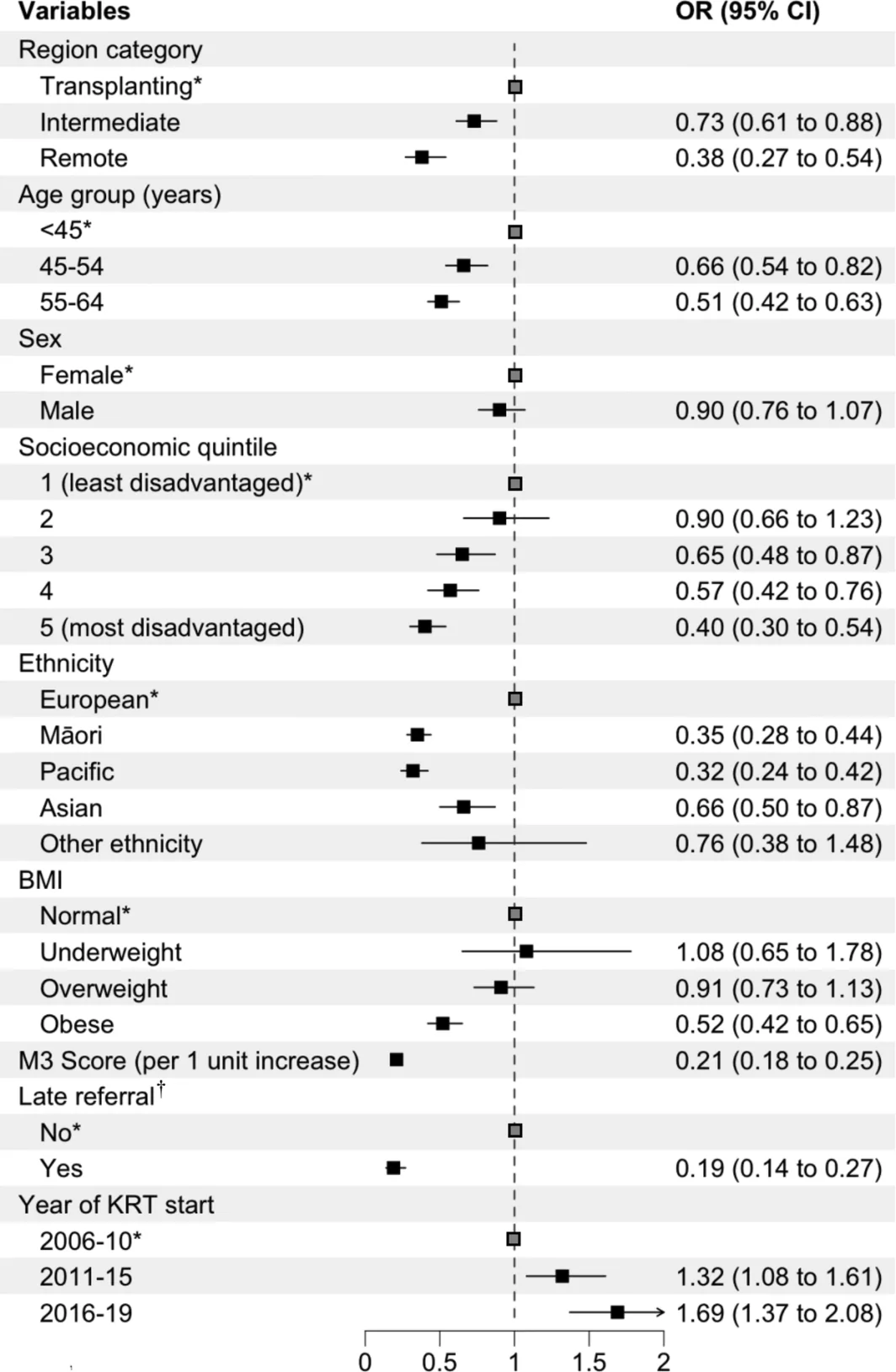
Forest plot of adjusted odds ratios for waitlisting or transplantation within 6 months of starting kidney replacement therapy, in people aged 2–64 years, New Zealand 2006–2019. * = Reference category, † = first nephrology assessment within 3 months of commencing KRT; BMI, body mass index; KRT, kidney replacement therapy; OR, odds ratio.

Predictors of waitlisting or living donor transplantation within 6 months of starting KRT, adjusting by region category, age group, sex, socioeconomic quintile, ethnicity, BMI category, multimorbidity, late referral and year category, are shown in Figure 5. A total of 5128 patients (aged 2–64 years) were included in the analysis. Table S4 provides unadjusted odds ratios (ORs) and separate results for waitlisting and living donor transplantation.

When compared to transplanting regions, living in an intermediate [OR 0.73 (95% CI 0.61–0.88)] or remote [OR 0.38 (95% CI 0.27–0.54)] region was independently associated with a lower likelihood of waitlisting or living donor transplantation within 6 months of starting KRT (Figure 5). Ethnicity was also an independent predictor; people of Māori [OR 0.35 (95% CI 0.28–0.44)], Pacific [OR 0.32 (95% CI 0.24–0.42)] and Asian [OR 0.66 (95% CI 0.50–0.87)] ethnicity were less likely to achieve the 6‐month transplantation target than those of European ethnicity. Māori and Pacific ethnicities were associated with disadvantage in both living donor transplantation and waitlisting, while Asian ethnicity was only associated with reduced access to living donor transplantation (Table S4).

Increasing age, socioeconomic disadvantage, obese BMI, increasing multimorbidity and late referral were also associated with a significantly lower likelihood of waitlisting or transplantation within 6 months of starting KRT. There was no significant difference by sex. Model fit statistics are outlined in Figure S4 and Table S5.

Figures S5–S8 provide further detail on changes over time in transplantation access, within region categories and ethnicity groups. In intermediate regions, there was a statistically significant improvement in the 6‐month waitlisting or transplantation outcome over the study period (Figures S5 and S6). However, patients living in remote regions were less likely to achieve the transplantation target than patients in transplanting regions across all time brackets of the study period, without statistically significant improvement over time, indicating widening disparity.

Figures S7 and S8 show temporal trends by ethnicity category. When compared to patients of European ethnicity, Māori and Pacific patients were disadvantaged in accessing timely waitlisting or transplantation across all time brackets.

## 4 DISCUSSION

This population‐based data linkage study explored geographic variation in the epidemiology of KRT in Aotearoa New Zealand and assessed the impact of residential location on access to kidney transplantation.

Our findings highlight significant spatial variation in the incidence of KRT, patient demographics and multimorbidity burden. Layering of disadvantage was observed. Health regions such as Tairawhiti, Northland, Counties Manukau and Lakes DHB recorded a high incidence of KRT (up to fourfold) when compared to other regions, in addition to substantially higher rates of socioeconomic disadvantage and multimorbidity among KRT recipients. We recommend that these regions are prioritized for investment in public health prevention and kidney care.

The current health reforms in New Zealand provide an opportunity to restructure current transplantation services to prioritize communities that are at greatest need and under‐served. In this study, KRT recipients of Māori and Pacific ethnicity were found to bear a greater burden of multimorbidity than other ethnicity groups. Furthermore, a higher proportion of the background population in remote regions are Māori (30%) than in intermediate (16%) and transplanting regions (10%; using 2013 Census data to approximate the midpoint of the study period).^17^ Our results demonstrate that people living most remotely from kidney care services are more likely to have complex care needs and require greatest investment in culturally appropriate models of care. The findings are consistent with the ‘inverse care law’ whereby the availability of optimal medical care tends to vary inversely with the need for it within the population served.^26^

We found that PD was the most frequent dialysis modality at 1 year after KRT commencement (47% of dialysis recipients), followed by in‐centre HD (43%) and home HD (10%). However, use of in‐centre HD is steadily increasing over time in New Zealand and has since become the predominant modality. In 2022, 64% of prevalent dialysis recipients were undertaking in‐centre HD, 24% PD and 12% home HD.^1^ It is important for clinicians to be aware of this trend and continue to counsel patients on home‐based dialysis and supportive care options where appropriate, providing individualized care.^27^ Further research is recommended to explore the factors contributing to the recent rise of in‐centre HD in New Zealand, including regional variation in KRT practices, patient travel time to dialysis centres and implications for capacity planning.

The ANZSN KPI of deceased donor waitlisting or transplantation within 6 months of KRT commencement (at age 2–64 years),^23^ had not previously been evaluated in New Zealand. We found that the KPI was achieved for 21% of patients starting KRT in New Zealand between 2006 and 2019, compared to a rate of 16% in Australia during 2022.^28^ KPI results in New Zealand improved significantly over the course of study period (Figure 5). Interactive maps, including DHB‐level KPI results, can be found here. These data can be used to inform targeted and equitable resource allocation for prevention and kidney care.

Living in a non‐transplanting region was found to be independently associated with disadvantage in timely access to waitlisting or transplantation. The reasons for this discrepancy are likely to be complex and vary between regions. As highlighted in our interactive maps, rates of late referral to a nephrology service ranged from 10% in Auckland DHB to over 20% in the remote DHBs. Late referral may reflect difficulties in obtaining primary care in remote areas and limits the time available for pre‐dialysis education and pre‐emptive transplant workup. We recommend process mapping of barriers to transplantation access in individual jurisdictions and implementation of region‐specific quality improvement strategies.

Achieving geographically equitable kidney health outcomes will require innovative models of nephrology service delivery in rural and remote regions of New Zealand. We recommend the development of a national strategy for improving kidney health outcomes in remote areas. Dynamic, appropriately resourced solutions that minimize travel burden for patients are needed. An effective strategy would address the social and cultural determinants of health, primary care and delivery of multidisciplinary nephrology services (including for chronic kidney disease, dialysis, transplantation and renal supportive care), with ongoing evaluation.

Previous reports have demonstrated that Māori and Pacific people are disadvantaged in accessing kidney transplantation, both pre‐emptively and after starting dialysis.^1^, ^2^ Our study has further quantified the factors contributing to this difference. Despite adjustment for region category, age group, socioeconomic status, BMI, multimorbidity and late referral, Māori and Pacific ethnicities were associated with disadvantage in both waitlisting and living donor transplantation. The discrepancy in access to living donor transplantation may at least partially be related to unmeasured confounders, such as family and friends from the same ethnicity group having higher rates of comorbidities that prevent them from becoming a donor. However, this does not account for the difference seen in access to the deceased donor waitlisting. Our results suggest that these ethnic inequities exist across all region categories and have not improved over the course of the study period (Figures S7 and S8).

Our findings provide further evidence of the need for the development of services that remove barriers to kidney transplantation for non‐European patients, particularly those of Māori and Pacific ethnicity. Recent qualitative research has provided recommendations from Māori patients, donors and whānau (extended family) for addressing racism during healthcare for kidney transplantation in New Zealand.^29^ These recommendations include workforce development, such as staff cultural awareness training and increasing the number of Māori staff and renal service leaders, and improved Māori cultural and spiritual support to navigate transplantation and kidney donation processes.

Amendments were made to the New Zealand Kidney Allocation Scheme in December 2022 to count all time spent on dialysis as waiting list time during deceased donor kidney allocation prioritization, rather than just considering time since waitlisting.^9^ This intervention may help to mitigate the effect of waitlisting delays for disadvantaged patients, with this group being prioritized for deceased donor transplantation once active on the list. Future re‐evaluation is recommended, including a comparison of the time from starting KRT to deceased donor transplantation among different demographic groups, before and after the policy change.

Since the study period, policy changes have also occurred to improve access to living donor transplantation in New Zealand. The Australian and New Zealand Paired Kidney Exchange (ANZKX) Program was established in October 2019, combining the previously separate kidney exchange programmes in both countries.^30^ ABO incompatible (ABOi) kidney transplants are performed at each transplant centre in New Zealand, but a reduction in ABOi transplants has been observed since 2019 with increases in kidney exchange activity.^30^ Following future data re‐linkage (including 2020 onwards), we recommend analysis of the impact of the ANZKX Program on transplantation access for disadvantaged populations in New Zealand.

The age‐standardized incidence of KRT in New Zealand was 9.6 (95% CI 9.4–9.8) cases per 100 000 population. In comparison, the age‐standardized incidence of KRT in Europe in 2017 was 14.8 cases per 100 000 (ranging from 9.8 in Finland to 22.3 in Greece) and has remained relatively stable over time.^31^ This may reflect a lower true incidence of kidney failure in New Zealand, or a higher local proportion of patients being managed with renal supportive care instead of KRT. Further studies capturing the renal supportive care group (such as from death certificates) would be useful to compare kidney failure management practices within New Zealand and internationally.

Limitations of this study include the use of domicile‐level data to estimate socioeconomic status and rurality. A high degree of variation exists within each domicile area, which was unable to be accounted for in our analyses due to the use of de‐identified patient data. Although total response ethnicity was assumed in the analyses and population data, multiple ethnicity categories were only recorded in ANZDATA for 16 of the 7704 patients. It is therefore likely that ethnicity categories were missed during ANZDATA data entry and under‐represented in our results. This could be further explored through data linkage to national ethnicity collections and comparison with ANZDATA.

To analyse access to transplantation, we used the ANZSN KPI of deceased donor waitlisting or living donor transplantation within 6 months of KRT commencement. Evaluation of subsequent waiting list dynamics or the achievement of transplantation was outside the scope of this study and has been explored elsewhere.^32^ Further causal analysis of transplantation outcomes, including effect modification using directed acyclic grafts and additional interaction terms, may be valuable. We also recommend qualitative evaluation of the additive effect of these factors on patients' lived experiences and outcomes.

## 5 CONCLUSION

This large population‐based study has identified populations that are currently disadvantaged in accessing kidney transplantation, providing a foundation for targeted strategies to improve service provision and transplant allocation. Geographic information system mapping provides opportunities for advanced spatiotemporal modelling to assist health service planning at a local and national level.

There is marked variation in the burden and socioeconomic profile of kidney failure across Aotearoa New Zealand. Living in a non‐transplanting region and non‐European ethnicity were independently associated with disadvantage in accessing kidney transplantation. The establishment of a national health service, Te Whatu Ora – Health New Zealand, provides opportunities to target resources for prevention and treatment of kidney failure towards populations at greatest need.

## FUNDING INFORMATION

This project was supported by funding from the Ross Bailey Nephrology Trust. JMB is supported by a National Health and Medical Research Council (NHMRC) Postgraduate Scholarship (ID 2031401). The research presented in this publication was also supported in part by funding received under the Australian Academy of Science's Douglas and Lola Douglas Scholarship. The funding organizations had no role in the design and conduct of the study; collection, management, analysis and interpretation of the data; preparation, review or approval of the manuscript.

## CONFLICT OF INTEREST STATEMENT

The authors have no conflicts of interest to declare.

## Supporting information


Figure S1.
