# Supplementary material for: Geographic variation in kidney failure and transplantation in Aotearoa New Zealand: A population‐based data linkage study
Source: Nephrology (Carlton). 2024 Oct 28;29(12):941–54. doi: 10.1111/nep.14409 (PMC13546947; doi:10.1111/nep.14409)
Supplement: Supplementary file 1 [file NEP-29-941-s001.docx]

*Supplementary Figure 1. Ethnicity, socioeconomic index and rurality of patients starting KRT from 2006-2019, compared to the general New Zealand population (Census 2018)*

Percentage of population in each ethnicity category

|  | European | Māori | Pacific | Asian | Middle Eastern/Latin American/African | Other ethnicity |
| --- | --- | --- | --- | --- | --- | --- |
| KRT cohort | 39 | 31 | 21 | 8 | 1 | 0.3 |
| General NZ Population | 62.1118 | 15.0843 | 7.09849 | 13.3097 | 1.33097 | 1.06477 |

Socio-economic decile*:

Percentage of population in each quintile

|  | 1-2 | 3-4 | 5-6 | 7-8 | 9-10 |
| --- | --- | --- | --- | --- | --- |
| KRT cohort | 9 | 12 | 16 | 22 | 41 |
| General NZ Population | 19.2 | 19.5 | 19.8 | 20.4 | 21.1 |

Rurality^†^:

Percentage of population living in each rurality category

|  | U1 | U2 | R1 | R2 | R3 |
| --- | --- | --- | --- | --- | --- |
| KRT cohort | 64 | 18 | 11 | 6 | 1 |
| General NZ Population | 63 | 18 | 12 | 6 | 1 |

KRT = kidney replacement therapy, NZ = New Zealand.

*NZDep2018: 9-10 = most disadvantaged, 1-2 = least disadvantaged.

^†^Geographic Classification for Health: R3 = most rural, U1 = most urban.

*Supplementary Table 1. Correlation between composite comorbidity indices*

| **Composite comorbidity indices** | **Spearman’s rho (r_s_)** | **Interpretation[1]** | **P value** |
| --- | --- | --- | --- |
| ANZDATA comorbidity count and NMDS* comorbidity count | 0.71 | Strong correlation | <0.001 |
| ANZDATA comorbidity count and CCI | 0.65 | Strong correlation | <0.001 |
| CCI and M3 Multimorbidity Index | 0.78 | Strong correlation | <0.001 |

* Within the 5 years prior to commencement of KRT

ANZDATA = Australia and New Zealand Dialysis and Transplant Registry

NMDS = National Minimum Dataset (hospital admissions)

CCI = Charlson Comorbidity Index

*Supplementary Figure 2. Comparison of data sources - proportion of patients starting KRT in each District Health Board with one or more recorded comorbidities*, 2006-2019*

Proportion of incident KRT recipients

|  | ANZDATA | NMDS ICD-10-AM codes |
| --- | --- | --- |
| Northland | 0.817 | 0.751 |
| Waitemata | 0.735 | 0.658 |
| Auckland | 0.768 | 0.723 |
| Counties Manukau | 0.802 | 0.772 |
| Waikato | 0.764 | 0.699 |
| Lakes | 0.836 | 0.78 |
| Bay of Plenty | 0.73 | 0.669 |
| Tairawhiti | 0.766 | 0.726 |
| Hawke's Bay | 0.742 | 0.713 |
| Taranaki | 0.754 | 0.688 |
| Midcentral | 0.605 | 0.542 |
| Whanganui | 0.723 | 0.67 |
| Capital and Coast | 0.637 | 0.578 |
| Hutt Valley | 0.668 | 0.624 |
| Wairarapa | 0.716 | 0.687 |
| Nelson Marlborough | 0.57 | 0.516 |
| West Coast | 0.5 | 0.486 |
| Canterbury | 0.594 | 0.532 |
| South Canterbury | 0.625 | 0.661 |
| Southern | 0.619 | 0.557 |

KRT = kidney replacement therapy

ANZDATA = Australia and New Zealand Dialysis and Transplant Registry

NMDS = National Minimum Dataset

ICD-10-AM = International Classification of Diseases and Related Health Problems, Tenth Revision, Australian Modification

*Included comorbidities: diabetes mellitus, coronary artery disease, peripheral vascular disease, cerebrovascular disease, chronic lung disease, cancer.

*Supplementary Table 2. Comparison of comorbidities between ANZDATA and hospital admission dataset*

| **Comorbidity prior to KRT commencement** | **ANZDATA**  **n=7,704** | **NMDS***  **n=7,704** | **Overall agreement (%)** ± **SE** | **Kappa** ± **SE** |
| --- | --- | --- | --- | --- |
| Diabetes mellitus | 4,222 (55%) | 4,312 (56%) | 97 ± 0.2 | 0.94 ± 0.003 (very good agreement) |
| Coronary artery disease | 1,924 (25%) | 1,308 (17%) | 84 ± 0.4 | 0.53 ± 0.006 (moderate) |
| Peripheral vascular disease | 978 (13%) | 691 (9%) | 88 ± 0.4 | 0.37 ± 0.006 (fair) |
| Cerebrovascular disease | 764 (10%) | 363 (5%) | 93 ± 0.3 | 0.46 ± 0.006 (moderate) |
| Chronic lung disease | 1,066 (14%) | 567 (7%) | 87 ± 0.4 | 0.31 (fair) ± 0.005 |
| Cancer | 898 (12%) | 569 (7%) | 93 ± 0.3 | 0.57 ± 0.006 (moderate) |
| Comorbidity count ≥ 1 | 5,640 (73%) | 5,212 (68%) | 88 ± 0.4 | 0.71 ± 0.005 (good) |

*Within the 5 years prior to commencement of KRT

ANZDATA = Australia and New Zealand Dialysis and Transplant Registry

NMDS = National Minimum Dataset (hospital admissions)

*Supplementary Table 3. First observed outcome for incident KRT recipients (2006-2019)*

| **First observed outcome** | **Number** | **Percentage** |
| --- | --- | --- |
| Deceased donor waitlisting | 2,075 | 26.9 |
| Living donor transplantation without being waitlisted* | 313 | 4.1 |
| Death without prior waitlisting or transplantation | 3,258 | 42.2 |
| Censored^†^ | 2,058 | 26.7 |
| Total | 7,704 | 100 |

*52% of living donor transplants performed without patients being waitlisted were pre-emptive.

^†^Censored patients are those who were lost to follow up before experiencing an event or remained on dialysis at the end of the follow up period. Methods adapted from [2].

*Supplementary Figure 3. Time between starting kidney replacement therapy and deceased donor waitlisting (years), by region category in New Zealand, 2006-2019*

KPI = key performance indicator (deceased donor waitlisting or transplantation within six months of KRT commencement, for people aged 2 to 64 years)

KRT = kidney replacement therapy.

*Supplementary Table 4. Logistic regression results: deceased donor waitlisting or living donor transplantation within six months of commencing kidney replacement therapy, in people aged 2 to 64 years, New Zealand 2006-2019.*

|  | **Unadjusted outcome: waitlisting or living donor transplantation** | | **Adjusted outcome: waitlisting** | | **Adjusted outcome: living donor transplantation** | | **Adjusted outcome: waitlisting or living donor transplantation** | |  |
| --- | --- | --- | --- | --- | --- | --- | --- | --- | --- |
|  | **OR** | **95% CI** | **OR** | **95% CI** | **OR** | **95% CI** | **OR** | **95% CI** |  |
| Region category | | | | | | | | |  |
| Transplanting | Reference | | | | | | | |  |
| Intermediate | 0.55^†^ | 0.48-0.64 | 0.92 | 0.77-1.11 | 0.78 | 0.59-1.02 | 0.73^†^ | 0.61-0.88 |  |
| Remote | 0.34^†^ | 0.25-0.44 | 0.62^†^ | 0.44-0.86 | 0.33^†^ | 0.18-0.60 | 0.38^†^ | 0.27-0.54 |  |
| Year |  |  |  |  |  |  |  |  |  |
| 2006-2010 | Reference | | | | | | | |  |
| 2011-2015 | 1.17 | 0.99-1.39 | 1.47^†^ | 1.19-1.81 | 1.02 | 0.74-1.40 | 1.32^†^ | 1.08-1.62 |  |
| 2016-2019 | 1.32^†^ | 1.12-1.57 | 1.87^†^ | 1.51-2.31 | 1.43* | 1.03-1.97 | 1.68^†^ | 1.37-2.08 |  |
| Age | | | | | | | | |  |
| <45 | Reference | | | | | | | |  |
| 45-54 | 0.59^†^ | 0.49-0.69 | 0.71^†^ | 0.58-0.88 | 0.67* | 0.48-0.91 | 0.66^†^ | 0.54-0.82 |  |
| 55-64 | 0.45^†^ | 0.38-0.53 | 0.62^†^ | 0.51-0.76 | 0.48^†^ | 0.34-0.66 | 0.51^†^ | 0.42-0.63 |  |
| Sex |  |  |  |  |  |  |  |  |  |
| Female | Reference | | | | | | | |  |
| Male | 1.05 | 0.91-1.20 | 0.84* | 0.71-0.99 | 1.10 | 0.84-1.44 | 0.90 | 0.76-1.07 |  |
| Socio-economic quintile |  |  |  |  |  |  |  |  |  |
| 1 (least disadvantaged) | Reference | | | | | | | |  |
| 2 | 0.74* | 0.57-0.95 | 1.09 | 0.80-1.50 | 0.89 | 0.61-1.30 | 0.90 | 0.66-1.23 |  |
| 3 | 0.45^†^ | 0.35-0.57 | 1.08 | 0.80-1.47 | 0.46^†^ | 0.30-0.69 | 0.65^†^ | 0.48-0.87 |  |
| 4 | 0.31^†^ | 0.25-0.40 | 0.91 | 0.68-1.23 | 0.46^†^ | 0.31-0.68 | 0.57^†^ | 0.42-0.76 |  |
| 5 (most disadvantaged) | 0.14^†^ | 0.11-0.18 | 0.67* | 0.50-0.91 | 0.22^†^ | 0.13-0.35 | 0.40^†^ | 0.30-0.54 |  |
| Ethnicity |  |  |  |  |  |  |  |  |  |
| European | Reference | | | | | | | |  |
| Māori | 0.15^†^ | 0.12-0.18 | 0.44^†^ | 0.35-0.56 | 0.22^†^ | 0.13-0.35 | 0.35^†^ | 0.28-0.44 |  |
| Pacific | 0.15^†^ | 0.12-0.19 | 0.44^†^ | 0.34-0.58 | 0.27^†^ | 0.15-0.45 | 0.32^†^ | 0.24-0.42 |  |
| Asian | 0.64^†^ | 0.50-0.81 | 1.02 | 0.77-1.34 | 0.23^†^ | 0.13-0.39 | 0.66^†^ | 0.50-0.87 |  |
| Other ethnicity | 0.91 | 0.50-1.60 | 1.19 | 0.60-2.28 | 0.27* | 0.06-0.80 | 0.76 | 0.38-1.48 |  |
| Body mass index |  |  |  |  |  |  |  |  |  |
| Underweight | 0.98 | 0.65-1.46 | 0.79 | 0.46-1.30 | 1.50 | 0.76-2.85 | 1.08 | 0.65-1.77 |  |
| Normal | Reference | | | | | | | |  |
| Overweight | 0.70^†^ | 0.59-0.84 | 0.91 | 0.72-1.12 | 0.90 | 0.66-1.24 | 0.91 | 0.73-1.13 |  |
| Obese | 0.26^†^ | 0.22-0.31 | 0.60^†^ | 0.48-0.74 | 0.50^†^ | 0.35-0.72 | 0.52^†^ | 0.42-0.65 |  |
| M3 Multimorbidity Index |  |  |  |  |  |  |  |  |  |
| 0 | Reference | | | | | | | |  |
| Per 1 point increase | 0.14^†^ | 0.12-0.17 | 0.26^†^ | 0.22-0.31 | 0.20^†^ | 0.15-0.28 | 0.21^†^ | 0.18-0.25 |  |
| Late referral^‡^ |  |  |  |  |  |  |  |  |  |
| No | Reference | | | | | | | |  |
| Yes | 0.22^†^ | 0.17-0.29 | 0.27^†^ | 0.20-0.37 | 0.13^†^ | 0.06-0.26 | 0.19^†^ | 0.14-0.27 |  |

*P < 0.05; ^†^P < 0.01; ^‡^ first nephrology assessment within 3 months of commencing KRT.

DD = deceased donor; LDTx = living donor transplantation.

*Supplementary Figure 4. Hosmer-Lemeshow plot for logistic regression model of waitlisting or transplantation within 6 months of starting kidney replacement therapy, in people aged 2 to 64 years, New Zealand 2006-2019.*

*Supplementary Table 5. Assessment of model fit*

| **Measure** | **Model 1 (waitlisting within 6 months)** | **Model 2 (living donor transplantation within 6 months)** | **Model 3 (waitlisting or living donor transplantation within 6 months)** |
| --- | --- | --- | --- |
| McFadden R^2^ test value | 0.24 | 0.35 | 0.33 |
| Area under the receiver operating characteristic (ROC) curve (c statistic) | 0.81 | 0.90 | 0.86 |
| Hosmer-Lemeshow test |  |  |  |
| - X-squared | 3.30 | 9.40 | 8.38 |
| - P value | 0.91 | 0.31 | 0.40 |

*Supplementary Figure 5. Trend in proportion of patients waitlisted or transplanted within 6 months of starting KRT, by region category, 2006-2019*

*Supplementary Figure 6. Forest plot of adjusted odds ratios for waitlisting or transplantation within six months of starting kidney replacement therapy, with interaction term between region category and year category*

^†^

* = reference category, ^†^ = first nephrology assessment within 3 months of commencing kidney replacement therapy; OR = odds ratio, BMI = body mass index.

P value for interaction term ([region category] x [year category]) = 0.09

If re-categorised into ‘transplanting’ and ‘non-transplanting’ (including intermediate and remote) region categories, P value for interaction term = <0.001.

*Supplementary Figure 7. Trend in proportion of patients waitlisted or transplanted within 6 months of starting KRT, by ethnicity category, 2006-2019*

*Supplementary Figure 8. Forest plots of adjusted odds ratios for waitlisting or transplantation within six months of starting kidney replacement therapy, with interaction term between ethnicity and year category*

* = reference category, ^†^ = first nephrology assessment within 3 months of commencing kidney replacement therapy; OR = odds ratio, BMI = body mass index.

P value for interaction term (ethnicity) x (year category) = 0.046.

‘Other ethnicity’ and ‘Asian ethnicity’ groups were combined in this analysis due to small patient numbers.

**Supplementary references**:

[1] Schober P, Boer C, Schwarte LA. Correlation Coefficients: Appropriate Use and Interpretation*.* *Anesth Analg*. 2018;126:1763-8.

[2] Sypek MP, Clayton PA, Lim W *et al*. Access to waitlisting for deceased donor kidney transplantation in Australia*.* *Nephrology (Carlton)*. 2019;24:758-76.
